# Accurate and rapid detection of *Fasciola hepatica* copro‐DNA in sheep using loop‐mediated isothermal amplification (LAMP) technique

**DOI:** 10.1002/vms3.455

**Published:** 2021-02-24

**Authors:** Siamak Amiri, Bahar Shemshadi, Saloomeh Shirali, Farnaz Kheirandish, Shirzad Fallahi

**Affiliations:** ^1^ Department of Pathobiology, Science and Research branch Islamic Azad University Tehran Iran; ^2^ Department of Parasitology and Mycology School of Medicine Lorestan University of Medical Sciences Khorramabad Iran; ^3^ Hepatitis Research Center Lorestan University of Medical Sciences Khorramabad Iran

**Keywords:** accurate, copro‐DNA, *Fasciola hepatica*, LAMP, rapid detection, sheep

## Abstract

Fascioliasis is a parasitic infection caused by *Fasciola* spp. in humans and animals. Despite significant advances in vaccination and new therapeutic agents, little attention has been paid to validate methods for the diagnosis of fascioliasis in animals. This study aimed to compare the loop‐mediated isothermal amplification (LAMP) technique with PCR assay for the diagnosis of *F. hepatica* in sheep. In this cross‐sectional study, 195 stool samples were collected from sheep for 3 months in Lorestan province, West of Iran. Specimens’ parasitological examination was performed by using the direct wet mount and formalin‐ether concentration method. After DNA extraction from the samples, molecular analysis was done using PCR and LAMP techniques based on the *Fasciola* ribosomal intergenic spacer (IGS) sequence. Of 195 specimens of sheep, 11 specimens were identified as *F. hepatica*‐positive infection by using microscopic, PCR and LAMP assays. Kappa agreement test results showed that there was a significant agreement between the results of microscopic examination diagnostic tests, PCR and LAMP (Kappa = 0.51–0.72 and *p* < .001). According to the results of chi‐square comparisons between parasite prevalence applying different techniques and variables of age, sex breed, and type of drinking water, there was no significant relationship (*p* ≥ .05). However, most of the infected sheep with *Fasciola* were 3‐ to 4‐year‐old females, of the Lori breed and consumed tap water. In many endemic areas, successful prevention and treatment of fascioliasis in animals depend on rapid and accurate diagnosis. Based on the results of the Kappa agreement, the significant agreement among the results of the microscopic examination, PCR and LAMP indicates the accuracy and reliability of these tests in the diagnosis of *F. hepatica* in sheep. However, molecular methods, especially the LAMP technique, are suggested because of their higher sensitivity and reliability for the diagnosis of *F. hepatica* even under field conditions.

## Graphical abstract

The liver flukes *F. hepatica* and *F. gigantica* are agents for fasciolosis that is a zoonotic parasitic disease. Bennett and Ijpelaar (*Journal of Agriculture Economy*, 2005, *56*, 135) estimated that *F. hepatica* causes annual economic loss by£40.4 million in the UK cattle industry. Fasciolosis not only infects the liver and biliary ducts, ectopic fasciolosis, but it may also infect the peritoneal cavity, lungs, subcutaneous tissue, lymph nodes, eye, and other locations. Fasciolosis can induce mortality and morbidity in the sheep and cattle industry. Many advances are observed in vaccination against *F. hepatica* but validated methods for the diagnosis of fascioliasis are still unknown. Fascioliasis is commonly diagnosed by fecal testing and finding parasitic eggs, bile, or fluid in the duodenum through examining direct stool and/or condensation techniques such as formalin‐ether and telman or Kato‐kats assays. Some serologic techniques such as Fas2‐ELISA, IFA, and IHA are applicable at all stages of the disease. These techniques have disadvantages such as the expertise of the test person and the number of parasite eggs in the stool sample. This study aimed to compare the loop‐mediated isothermal amplification (LAMP), one of the relatively new molecular techniques with PCR assay in the diagnosis of *F. hepatica* in sheep.

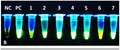

## 1 INTRODUCTION

*Fasciola hepatica (F. hepatica)*, known as liver fluke, is a parasitic trematode which is commonly observed in temperate climates. *F. hepatica* not only infects sheep and cattle but also infects horses, deer, buffalo and camelids (John et al., 2019). Flukes are commonly found in the liver and bile ducts of their definitive host, and causes acute and chronic diseases with clinical signs such as anaemia, liver dysfunction and weight loss. The liver flukes *F. hepatica* and *Fasciola gigantica* (*F. gigantica*) are the agents causing fasciolosis, a zoonotic parasitic disease with typical clinical signs of fever, nausea, a swollen liver, skin rashes and extreme abdominal pain (John et al., 2019). *Fasciola* not only infects the liver and biliary ducts, but it may also infect the peritoneal cavity, lungs, subcutaneous tissue, lymph nodes, eye and other locations. Fasciolosis can induce considerable mortality and morbidity in livestock (Hosseini‐Safa et al., 2019). According to the Centers for Disease Control and Prevention (CDC) reports *F. hepatica* is found in focal areas of more than 70 countries, in all continents except Antarctica. It is found in parts of Latin America, Europe, the Caribbean, Africa, the Middle East, Oceania and Asia. *F. gigantica* is found in fewer geographic regions (CDC, 2018). Bennett and Ijpelaar (2005) estimated that *F. hepatica* causes an annual economic loss of £40.4 million in the UK cattle industry. The prevalence of 0.1% to 91.4% was reported in various livestock in Iran. In past decades, the infection rates of livestock were higher in southern Iran, while the human disease has been mostly reported in Northern provinces especially in Rasht (Ashrafi, 2015; Badparva et al., 2009; Fallahi et al. 2016). Many advances are observed in vaccination against *F. hepatica*, but validated methods for the diagnosis of fascioliasis are still unknown. Fascioliasis is commonly diagnosed with fecal testing and finding parasitic eggs in stool, bile or fluid in the duodenum through wet mount and/or condensation techniques such as formalin‐ether and Telman or Kato‐Kats assays. Some serologic techniques such as Fas2‐enzyme‐linked immunosorbent assay (Fas2‐ELISA), immunofluorescence assay (IFA) and Indirect Hemagglutination Assay (IHA) are applicable at all stages of the disease. These techniques have disadvantages such as the examiner's expertise and the number of parasite eggs in the stool sample. The different molecular techniques are used for diagnosis and study of *Fasciola* including whole‐genome sequencing (WGS) and single nucleotide polymorphism (SNP), Random amplified polymorphic DNA (RAPD) and polymerase chain reaction‐restriction fragment length polymorphism (PCR‐RFLP; Hamoo et al., 2019). Loop‐mediated isothermal amplification (LAMP) is a simple, sensitive and inexpensive assay which allows rapid and high‐sensitive amplification of a small amount of DNA under isothermal conditions (typically at 63°C; Fallahi et al., 2014, 2015; Kheirandish et al., 2020; Mirahmadi et al., 2020; Mori et al., 2001; Soltani Tehrani et al., 2020; Valian et al., 2020). The LAMP has a specific reaction because it uses four specific primers [viz. F3, FIP (F1c + F2), BIP (B1c + B2) and B3] binding to six independent sites in the target sequence (Fallahi et al., 2020; Ghodsian et al., 2019; Hanifehpour et al., 2019; Parida et al., 2008). An appropriate selection of the target gene and the optimal design of turn‐back primers have successful LAMP result (Fallahi et al., 2017, 2018; Ghodrati et al., 2017; Hoa Le et al., 2012) and cause visible turbidity (Mori & Notomi, 2009). This technique is carried out using inexpensive equipment such as a regular water bath or a heating block, and its products are easily seen with the eye or visualized by the addition of fluorescent dyes such as SYBR green I, with or without using a UV lamp (Mori et al., 2001).

LAMP is a technique for the rapid diagnosis of a range of zoonotic Platyhelminthes, such as *F. hepatica* and *F. gigantica* (Ai et al., 2010), *Clonorchis sinensis* (Cai et al., 2010), *Schistosoma japonicum* (Xu et al., 2010), *Taenia* spp. (Nkouawa et al., 2009), *Paragonimus westermani* (Chen et al., 2011) and, recently, *Opistorchis viverrini* (Arimatsu et al., 2012). In many endemic areas, successful prevention and treatment of fascioliasis in animals depend on rapid and accurate diagnosis. The present study aimed to apply and compare the two molecular methods, LAMP and PCR, in the diagnosis of F. hepatica in sheep stool samples.

## 2 MATERIALS AND METHODS

### 2.1 Samples collection

Over 3 months, a total of 195 stool samples were collected from sheep in Lorestan province, West of Iran by stratified random sampling method. Lorestan province consisted of 11 cities, 29 districts and 85 villages. From each district (including different urban areas and villages) samples were collected by cluster sampling. The distribution of samples in each cluster has also been observed according to the animals grazing types. After sampling, the specimens were immediately transferred to the Parasitology Laboratory, Faculty of Medicine, Lorestan University of Medical Sciences, Khorramabad, West of Iran. Each sample was divided into two parts: one part for molecular analysis which was stored at −20°C until DNA extraction and the other one was used for routine parasitological experiments.

### 2.2 Direct stool examination

For parasitological examination, about 30 ml of normal saline (0.9% sodium chloride isotonic solution) was poured into the sampling container and placed at room temperature (20–22°C) for 30 min. The sample mixed with normal saline passed through the sieve to remove large stool particles from the sample. After completion of this step, the passed mixture was poured into an Eppendorf tube and centrifuged at 2,000 *g* for 4 min. A drop of sediment was deposited on the slide and examined directly at microscopic magnifications of 4, 10 and 40×.

### 2.3 Formalin ethyl acetate concentration

A few grams of fecal sample precipitate passed through the sieve in a test tube was mixed with approximately 9 ml of 10% formalin and incubated at room temperature for 30 min, and then 2–3 ml of ethyl acetate was added to each tube. The lids were blocked and each tube was shaken for 30 s. Then, in order to remove the gas produced in the tube, the tube cap was gently removed and the samples were centrifuged at 2,500 *g* for 4 min. Each tube consists of four layers (bottom to top: fecal sample sediment, formalin, debris and ethyl acetate respectively). The debris layer was removed by an applicator and then all the contents of the pipe except the sediment were discarded. From the resulting precipitate, a drop was placed on the slide and examined at microscopic magnifications of 4, 10 and 40 ×.

### 2.4 DNA extraction

A specific DNA extraction kit (MBST Co.) was used for extracting the DNA from stool samples according to the kit manufacturer's instructions. To break the *Fasciola* eggshell and facilitate the DNA extraction before extraction, the deposition of each stool sample that was passed through the sieve in the first step was sonicated at 90 volts and 5 s for five times. Then, 300 µl of the sample was poured into 1.5 µl microtubes and DNA extraction was performed. Eggs isolated from the uterus of several *Fasciola* worms that were isolated from the liver of infected slaughterhouse animals and identified and confirmed morphologically were used as a positive control for sonication and extraction procedures. After each step of sonication for 5 s, a drop of the sample was placed on the slide to check for egg breakage. Also, after the extraction step on the control samples, the extracted DNA concentration was measured using a Nanodrop machine (Thermo Scientific™ NanoDrop™ 2000/2000c Spectrophotometers, Thermo Fisher Scientific).

### 2.5 PCR assay

The primers used in the LAMP technique targeting the ribosomal intergenic spacer (IGS) region of the *F. hepatica* genome were adapted from the study of Ai et al. (2010). The F3 and B3 external primers of the LAMP technique were also used in the PCR assay. PCR was conducted in a final volume of 25 μl containing 12.5 µl of master mix (Ampliqon), 2 µl of F and R primers, 2 µl of extracted DNA and 8.5 µl of distilled water. Reactions were amplified in a thermocycler (Bio‐Rad) as follows: initial denaturation at 94°C for 5 min; afterward 35 cycles of denaturation at 94°C for 1 min, annealing at 55°C for 45 s and extension at 72°C for 1 min. The final extension was performed at 72°C for 5 min. Negative‐control samples (sterile water) were included in all PCRs. The PCR products were electrophoresed in a 1% TBE (Tris base–boric acid–EDTA) agarose gel and stained with DNA safe stain solution (1 μg/ml). The PCR amplification is expected to yield 220 bp products for a positive reaction.

### 2.6 LAMP reaction solution and optimization

Optimization of LAMP with specific primers was conducted using three different cycling temperatures 61°C, 63°C and 65°C and two different concentrations of Mg^2+^, 8 and 10 mM. Another optimization was conducted using the same protocol but with three different amplification times, 30, 45 and 60 min, at 63°C with 8 mM Mg^2+^ concentration. All LAMP products were stored at 4°C for further analyses.

The LAMP assay was carried out in a total of 25 µl reaction mixture containing 1 µl of template DNA, 40 pmol of primers FIP and BIP, 20 pmol each of primers LF and LB, 5 pmol each of primers F3 and B3, 8 U of *Bst* DNA polymerase (New England Biolabs), 1.4 mM deoxynucleoside triphosphates (dNTP), and 2× reaction buffer (1.6 M betaine (Sigma‐Aldrich), 40 mM Tris–HCl (pH 8.8), 20 mM KCl, 20 mM (NH_4_)_2_SO_4_, 16 mM MgSO_4_, and 0.2% Tween 20). The template DNA was omitted in one reaction as the negative control. The mixture was incubated at 61–65°C for 60 min and then heated at 80°C for 10 min to terminate the reaction. The LAMP products were analysed by naked eyes using the turbidity of magnesium pyrophosphate. Also, 1 µl of 1/10 dilution of SYBR Green I (Invitrogen lot: 49743A) was added in the reaction tubes to visually inspect LAMP amplicons, and afterward fluorescent signals of the reaction mixtures were observed using UV Image system (UVItec). To avoid carryover contamination with LAMP amplicons by opening the tubes, the reaction tubes were centrifuged at 1,300 *g* for 3 min and then were frozen at −20°C for 10 min, before adding the SYBR Green I.

### 2.7 The analytical sensitivity of the LAMP and PCR

The analytical sensitivity of LAMP and PCR assay was determined by preparing the serial numbers of *F. hepatica* eggs in PBS (0, 1, 5, 10, 20, 50 and 100 eggs/ml) and extracting the DNA as described above. All experiments on the dilution series with the LAMP and PCR methods were performed in duplicate. Negative control was included in all molecular assays.

### 2.8 Sequencing PCR products for final approval

The PCR products of external primers F3 and B3 were sequenced to confirm the accuracy of the PCR and the LAMP assays.

### 2.9 Statistical analysis

The Kappa agreement test was used to determine the degree of agreement between the methods. Statistical analysis was done by using the Chi‐square test and SPSS software version 19. A *p‐value* less than .05 (typically ≤.05) was considered statistically significant.

## 3 RESULTS

### 3.1 Demographic and contextual characteristics of the studied animals

In the present study, 19.5% and 13.8% of the animal samples were collected from Khorramabad and Aligodarz cities respectively. About 48.2% of sheep examined were from 50 to 99 herds. Most of the sheep studied had traditional grazing (79.5%), were female (62.6%) and in the age group of 3–4 years (39.5%). Other demographic and contextual characteristics of the studied animals are listed in Table 1.

**TABLE 1:** Demographic and contextual characteristics of the studied animals based on the results of PRC and LAMP assays

Variables	Value	Sheep number	LAMP result		*p* value	PCR result		*p* value
		*N* (%)	Positive *N* (%)	Negative *N* (%)		Positive *N* (%)	Negative *N* (%)	
Area of sampling	Khorramabad	38 (19.5)	1 (2.6)	37 (97.4)	0.580	1 (2.6)	37 (97.4)	0.517
	Aligoudarz	27 (13.8)	1 (3.7)	26 (96.3)		0 (0.0)	27 (100)	
	Poledokhtar	22 (11.3)	0 (0.0)	22 (100)		0 (0.0)	22 (100)	
	Delfan	22 (11.3)	2 (9.1)	20 (90.1)		2 (9.1)	20 (90.1)	
	Kouhdasht	18 (9.2)	1 (5.6)	17 (94.4)		1 (5.6)	17 (94.4)	
	Other	68 (34.9)	6 (8.8)	62 (91.2)		3 (4.4)	65 (95.6)	
Number of sheep per herd	<50	60 (30.8)	4 (6.7)	56 (93.3)	0.256	1 (1.7)	59 (98.3)	0.115
	50–99	94 (48.2)	7 (7.4)	87 (92.6)		6 (6.4)	88 (93.6)	
	>=100	41 (21.0)	0 (0.0)	41 (100)		0 (0.0)	41 (100)	
Grazing type	Traditional	155 (79.5)	11 (7.1)	144(92.9)	0.124	7 (4.5)	148(95.5)	0.348
	Industrial	40 (20.5)	0 (0.0)	40 (100)		0 (0.0)	40 (100)	
Gender	Male	73 (37.4)	5 (6.8)	68 (93.2)	0.750	3 (4.1)	70 (95.9)	1.000
	Female	122 (62.6)	6 (4.9)	116(95.1)		4 (3.3)	118(96.7)	
Age	1–2	72 (36.9)	3 (4.2)	69 (95.8)	0.252	2 (2.8)	70 (97.2)	0.191
	3–4	77 (39.5)	7 (9.1)	70 (90.9)		5 (6.5)	72 (93.5)	
	5–6	46 (23.6)	1 (2.2)	45 (97.8)		0 (0.0)	46 (100)	
Type of water consumed	Well, Spring, River water	32 (16.4)	3 (9.4)	29 (90.6)	0.393	1 (3.1)	31 (96.9)	1.000
	Tap water	163 (83.6)	8 (4.9)	155 (95.1)		6 (3.7)	157 (96.3)	

### 3.2 Direct stool examination

**FIGURE 1:**
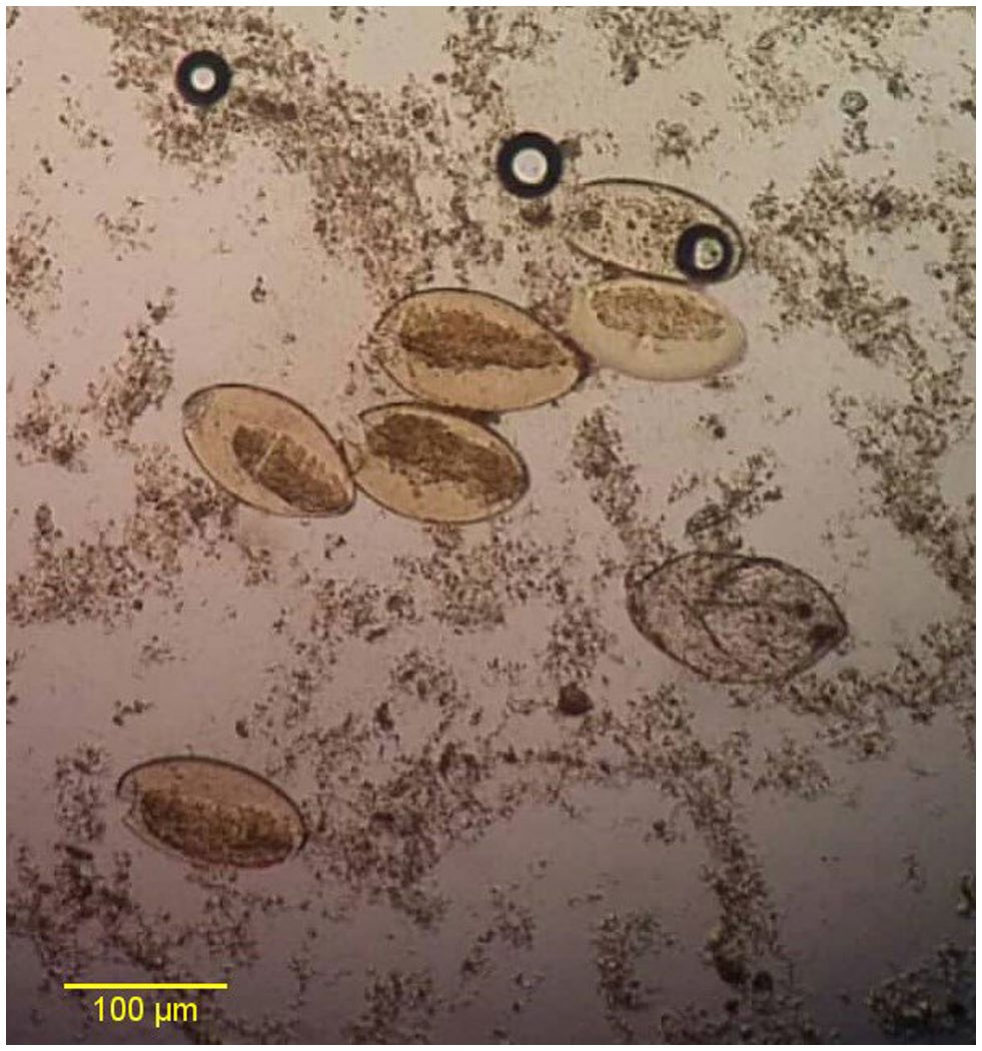
Microscopic image of *Fasciola hepatica* eggs (×40 objective lens)

Of 195 stool samples collected from sheep 4 samples (2.1%) were found to be infected with *Fasciola* spp. eggs by parasitology assay and microscopic examination (Figure 1).

### 3.3 Positive samples collection and DNA extraction

The parasites isolated from the liver were V‐shaped at their ends and had a length‐to‐width ratio of 2.6 to 2.8, which were in the length‐to‐width range of *F. hepatica*. The DNA concentrations of egg samples harvested from the liver of the infected animals were 145–180 ng/µl and the absorbance of 260 to 280 ratios was read between 1.9 and 2.1.

### 3.4 PCR assay

**FIGURE 2:**
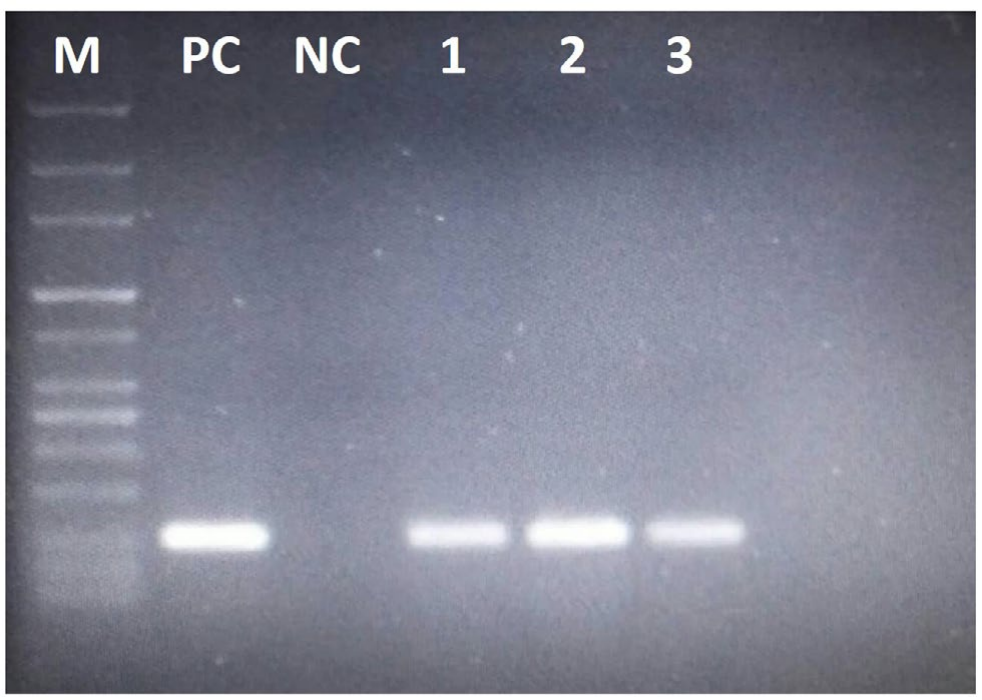
Results of the *Fasciola hepatica* IGS PCR on 1% agarose gel electrophoresis. M, Molecular weight 100bp marker; PC, Positive control; NC, Negative control; 1–3 positive samples for *Fasciola hepatica*

Electrophoresis of PCR products on 1% agarose gel stained with DNA safe stain revealed that among 195 DNA samples extracted from sheep stool samples, 7 specimens (3.6%) were diagnosed positive with *F. hepatica* (Figure 2).

### 3.5 Sequence analysis

Final products of the seven PCR‐positive samples using F3 and B3 external LAMP primers were sequenced, which showed 100% identification and 98%–99% query coverage with the IGS region of the *F. hepatica* genome at Gen Bank (Accession No. FhCM1‐*F. hepatica* (GU903890)). This result showed the final confirmation of *F. hepatica* eggs in which the LAMP reaction was positive.

### 3.6 LAMP reaction

**FIGURE 3:**
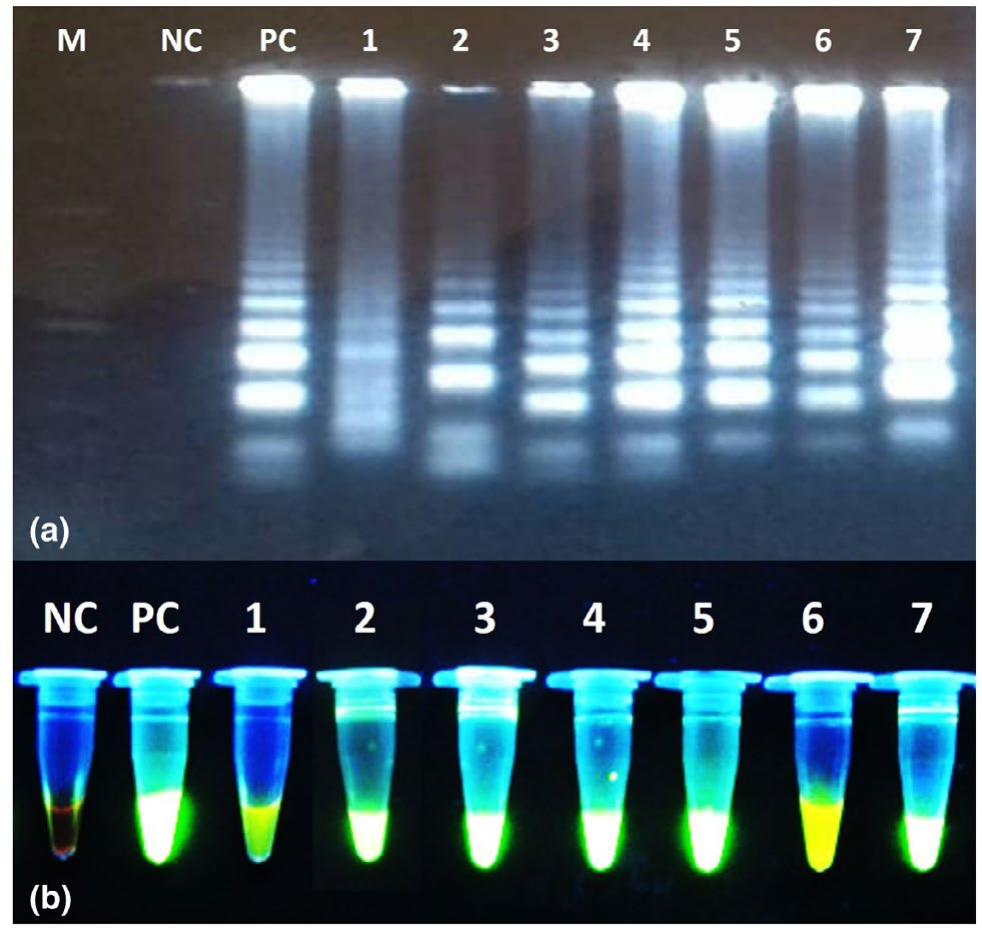
Results of the *Fasciola hepatica* IGS LAMP on 1.5% agarose gel electrophoresis (a) and under UV light (B). M, Molecular weight 100bp marker; NC, Negative control; PC, Positive control; 1–7 positive samples for *F. hepatica*

After optimization, the optimum conditions for LAMP reaction with specific primers were achieved using 8 mM Mg^2+^, at 63°C for 60 min. Upon completion of the LAMP reaction and the addition of SYBR GREEN I fluorescent dye to the reaction tubes, 11 tubes (5.6%) showed green fluorescence indicated a positive result of *F. hepatica* infection (Figure 3a,b).

### 3.7 The analytical sensitivity of the LAMP and PCR

The analytical sensitivities of LAMP and PCR assays were evaluated against the preparation of the serial numbers of *F. hepatica* egg from 0 to 100 eggs/ml. The detection limits of the LAMP and PCR assays were 1 and 10 eggs of *Fasciola*, respectively, showing the higher sensitivity of the LAMP technique compared with PCR (Table 2).

**TABLE 2:**
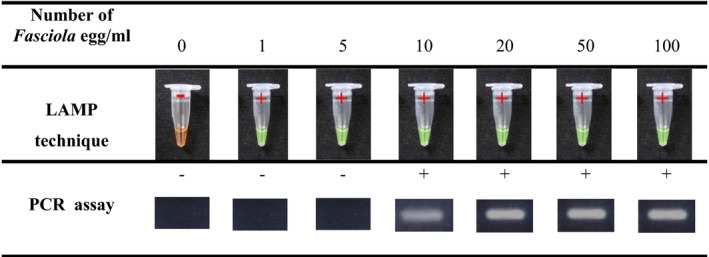
The analytical sensitivity of LAMP and PCR assays against the serial numbers of <em>Fasciola</em> egg from 0 to 100 eggs/ml

### 3.8 The agreement between diagnostic tests

Kappa test results showed a significant agreement between the results of PCR and microscopic assays (Kappa = 0.72 and *p* < .001). So that four positive samples identified by microscopic examination were also positive in the PCR method. Also, the results of the Kappa agreement test showed that there is a significant agreement between the results of LAMP and microscopic assays (Kappa = 0.51 and *p* < .001). Positive samples detected by the microscopic method which they were also detected positively by the LAMP technique. Also, seven negative samples detected by microscopy had a positive result in the LAMP technique. However, based on the results of the Kappa test, there was a significant agreement between the results of PCR and LAMP diagnostic tests (Kappa = 0.65 and *p* < .001). One of the positive samples detected by the PCR method had a negative result in the LAMP technique (Table 3).

**TABLE 3:** Comparison of the results of LAMP technique with microscopic examination and PCR assay for the detection of *Fasciola hepatica*

Diagnostic assay	LAMP technique		Total	Kappa CO	*p* value
	Negative *N* (%)	Positive *N* (%)			
Microscopic examination					
Negative *N* (%)	184 (94.4)	7 (3.6)	191 (97.9)	0.51	<0.001
Positive *N* (%)	0 (0.0)	4 (2.1)	4 (2.1)		
Total	184 (94.4)	11 (5.6)	195 (100)		
PCR assay					
Negative *N* (%)	183 (93.8)	5 (2.6)	188 (96.4)	0.65	<0.001
Positive *N* (%)	1 (0.5)	6 (3.1)	7 (3.6)		
Total	184 (94.4)	11 (5.6)	195 (100)		

### 3.9 Relationship between the prevalence of *Fasciola* infection and demographic characteristics of the studied animals

According to the results of the chi‐square test, there was no significant difference between parasite prevalence using the LAMP technique in different cities (*p* = .580). However, *Fasciola* had the highest prevalence in Delfan town (9.1%) and the lowest prevalence observed in Poledokhtar (0.0%). Also, according to the results of the Chi‐square test, there was no significant difference between *Fasciola* prevalence and LAMP technique in herds with different numbers (*p* = .256). However, the highest prevalence of parasites was seen in 50 to 99 (7.4%) herds and the lowest prevalence was observed in herds with livestock numbers above 100 (0.0%). According to the same test, although all 11 sheep infected with *F*. *hepatica* (7.1%) were in the traditional grazing group, no significant difference was found between the two groups with traditional and industrial grazing (*p* = .124).

The results of the Chi‐square test showed that there was no significant relationship between *F. hepatica* infection based on LAMP technique and age, sex, breed and type of drinking water (*p* ≥ .05). The mean age was 3–4 years (9.1%), female (4.9%), Lori breed (6.3%) and tap water consumers (4.9%). According to the results of the comparison of chi‐square ratios between parasite prevalence by PCR technique in different cities, there was no significant difference (*p* = .517). Also, according to the results of the Chi‐square test, there was no significant difference between *Fasciola* prevalence and PCR in herds with different numbers (*p* = .115). However, the highest prevalence of parasites in herds 50 to 99 (6.4%), and the lowest prevalence was observed in herds with livestock numbers above 100 (0.0%). According to the results of this test, although all seven infected sheep detected by PCR (4.5%) were in the traditional grazing group but no significant difference was detected between the traditional and industrial grazing groups (*p* = .348). Chi‐square test results showed that there was no remarkable relationship between *F. hepatica* infection based on PCR technique and variables of age, sex, breed and type of drinking water (*p* ≥ .05). The mean age was 3–4 years (6.5%), female (3.3%), Lori breed (3.8%) and tap water (3.7%; Table 1).

## 4 DISCUSSION

Although the clinical and economic importance of fasciolosis has been known for centuries, existing diagnostic tests are not very suitable for diagnosing infection in animals. The choice of the type of diagnostic method is usually influenced by the purpose of the study. The gold standard approaches and/or experimental settings have mainly been used to evaluate the diagnostic test and their limitations (Mazeri et al., 2016). Nonetheless some advances in establishing immunologic techniques, these tests lack high sensitivity and/or specificity in diagnosis. Even so, there is considerable potential for antigen detection tests, but these tests are not sufficiently evaluated in field conditions. Stool egg count and coproantigen testing have been suggested as appropriate methods for diagnosing drug efficacy/resistance. Early diagnosis of *F. hepatica* infection is provided by serological methods, but circulating antibodies may remain in the blood for several months after successful treatment. Therefore, serology does not always measure the current infection but only the exposure to the parasite (Immaculata Arifin et al., 2016; Salimi‐Bejestani et al., 2005). Furthermore, it seems the advanced nucleic acid‐based methods such as PCR and LAMP to be the most promising for the diagnosis of current infection in naturally infected sheep with the same or increased susceptibility compared with microscopic examination and coproantigen ELISA (cELISA) detects (Alvarez Rojas et al., 2014; Davies Calvani et al., 2018; Immaculata Arifin et al., 2016).

In the current study, the routine parasitology examination (direct wet mount and concentration method), PCR and LAMP techniques were compared together for the diagnosis of *F. hepatica* in stool samples of sheep. Based on the acquired results, the most positive cases for *F. hepatica* were diagnosed by LAMP and PCR assays (11 and 7 cases) respectively. Using the parasitology examination, only 4 (2.1%) stool samples were diagnosed positive for *F. hepatica*. These findings confirm that molecular techniques are much more capable of detecting *Fasciola* infection in animals (Table 1).

In the study by Immaculata Arifin and colleagues, the outcomes of fecal egg count (FEC), serology and coproantigen ELISA (cELISA) were compared with the performance of PCR and LAMP in the diagnosis of *F. hepatica* from naturally infected cattle and sheep. The *F. hepatica* eggs were observed in 28 animals, while coproantigen and specific anti‐*F. hepatica* antibodies were detected in 36 and 53 animals respectively. Contrary to the results of the current study, the PCR and LAMP assays detected only three and six positive samples, respectively, which they concluded that PCR and LAMP are highly specific, but they both had poor sensitivity compared with FEC and cELISA (Immaculata Arifin et al., 2016). In another study conducted by Martínez‐Valladares et al., a LAMP assay was developed to improve the diagnosis of *Fasciola* spp. in the feces of sheep. Contrary to the results of the present study, in their survey, the LAMP technique showed similar results with the standard PCR using the outer primers of the LAMP reaction (a detection limit of 10 pg in both assays). Based on their results, both techniques diagnosed the fasciolosis during the first week post‐infection in experimentally infected sheep before treatment with triclabendazole and even on Day 30 post‐treatment. They concluded considering the LAMP assay solves the drawbacks of the standard PCR including time consuming, variable sensitivity and the need for expensive thermal cycler and UV transilluminator gel documentation could be a good alternative to conventional diagnostic methods to detect *F. hepatica* in feces (Martínez‐Valladares et al., 2016). The difference between the results of the above two studies and the current study may be due to different test conditions, the presence of reaction inhibitors in fecal samples and differences in the type of reagents and enzymes used in PCR and LAMP reactions.

Ayaz et al. (2014) compared the PCR and microscopy for the diagnosis of *F. hepatica* in some buffaloes and cattle. Their results showed that PCR was a more sensitive method of *Fasciola* diagnosis than microscopy. In another study, the performance of five diagnostic tests including gall bladder egg count, fecal egg counting, a commercially available coproantigen ELISA, an in‐house serum excretory/secretory antibody ELISA and routine abattoir liver inspection were evaluated and compared for *F. hepatica* infection in naturally infected cattle. Their results provide evidence to suggest that unlike antibody ELISA, the coproantigen ELISA does not cross‐react with *Calicophoron daubneyi* rumen fluke parasites. Moreover, they reported that fecal egg counting has been shown to still be a valuable tool in the diagnosis of current *F. hepatica* infections, but one has to bear in mind that it is a weak test during periods where recent infections are expected. The coproantigen ELISA is a comparison test that can be used throughout the year. They also reported that in‐house ES antigen ELISA showing that while being a valuable test, its sensitivity and specificity estimates are lower in the field setting than previously reported (Mazeri et al., 2016). In a study by Ai et al. (2010), the LAMP technique was applied and compared with conventional PCR for rapid identification and differentiation of *F. hepatica* and *F. gigantica*. The results of their study showed higher sensitivity (approximately 10^4^ times) than using the LAMP technique, even higher than the sensitivity obtained in the current study by the PCR method in the diagnosis of *Fasciola* spp.

According to the Kappa agreement test results, there was a significant agreement among the results of microscopic examination, PCR and LAMP (Kappa = 0.51 –0.72 and *p* < .001; Table 3). This result revealed that more sensitive methods could be used or a combination of several methods simultaneously can be applied for the *Fasciola* infection diagnosis in livestock. Comparisons between parasite prevalence (achieved by different techniques) and variables of the sampling area, age, sex, breed and type of drinking water by the chi‐square test revealed that there was no significant relationship (*p* ≥ .05). However, most of the sheep infected with *Fasciola* were from Delfan town, females, of the age of 3–4 years, of the Lori breed and consumed tap water (Table 1). Surface waters such as river water and springs have a higher potential for transmitting contamination to livestock as well as humans due to the possibility of the presence of *Fasciola* vectors snails than well water or tap water. Drinking water carrying floating metacercariae originated from natural water collections inhabited by lymnaeid snail vectors in the field in an endemic area should be considered as one of the possible routes of animal infection (Mas‐Coma et al., 2018). All animals studied had a history of antiparasitic drug consumption. Due to the low prevalence of parasites (11 cases) in this study, we were not allowed to use multivariate modelling to "investigate the determinants of parasites" and therefore based only on univariate correlations. The cut‐off for a significance test is 0.05.

Overall, our study offers new insight into the performance of some diagnostic tests available for the diagnosis of *F. hepatica* infections in a sheep population supposed to be demonstrative of the field condition. LAMP technique can be a valuable assay in monitoring and understanding the changing epidemiology of *F. hepatica* as well as evaluating farm health plans by knowing its flaws and their ability of adjustment. The PCR assay is a comparable test that can be used throughout the year, with acceptable sensitivity and specificity. Parasitology examination has been shown to still be a valuable assay in the diagnosis of *F. hepatica* infections, although it is a weak test in periods where recent infections are expected to occur. Fascioliasis control is a worldwide challenge, hence the qualitative and quantitative evaluation of existing diagnostic tests, as well as the development of better field trials is mandatory.

## CONFLICT OF INTERESTS

None.

## AUTHOR CONTRIBUTIONS

**Siamak Amiri:** Investigation; Methodology; Project administration. **Bahar Shemshadi:** Conceptualization. **Saloomeh Shirali:** Conceptualization; Visualization. **Farnaz Kheirandish:** Conceptualization; Supervision; Writing‐original draft; Writing‐review & editing.

## ETHICAL STATEMENTS

The authors confirm that the ethical policies of the journal, as noted on the journal's author guidelines page, have been adhered to. The study was approved by the ethical committee of the Science and Research branch, Islamic Azad University, Tehran, Iran.

### Peer Review

The peer review history for this article is available at https://publons.com/publon/10.1002/vms3.455.
